# Saving Energy Using the Modified Heuristic Algorithm for Energy Saving (MHAES) in Software-Defined Networks

**DOI:** 10.3390/s23239581

**Published:** 2023-12-02

**Authors:** Péter András Agg, Zsolt Csaba Johanyák

**Affiliations:** Department of Information Technologies, GAMF Faculty of Engineering and Computer Science, John von Neumann University, 6000 Kecskemét, Hungary; agg.peter@nje.hu

**Keywords:** SDN, SDN networks, energy saving, compression TCAM, end host-aware, traffic-aware, rule placement, MHAES, algorithm, Python

## Abstract

Energy consumption is a significant concern in daily life, often neglected in terms of cost and environmental impact. Since IT networks play an essential role in our daily routines, energy-saving in this area is crucial. However, the implementation of energy efficiency solutions in this field have to ensure that the network performance is minimally affected. Traditional networks encounter difficulties in achieving this goal. Software-Defined Networks (SDN), which have gained popularity in the past decade, offer easy-to-use opportunities to increase energy efficiency. Features like central controllability and quick programmability can help to reduce energy consumption. In this article, a new algorithm named the Modified Heuristic Algorithm for Energy Saving (MHAES) is presented, which was compared to eight commonly used methods in different topologies for energy efficiency. The results indicate that by maintaining an appropriate load balance, one can save more energy than in case of using some other well-known procedures by applying a threshold value based on forecast, keeping only a minimal number of nodes in an active state, and ensuring that nodes not participating in packet transmission remain in sleep mode.

## 1. Introduction

Software-Defined Networks (SDN) [1,2] are regarded as valuable solutions in computer networks and are gaining increasing prominence in modern times. The idea of SDN originated from the fact that the optimal configuration of the network posed numerous difficulties and required substantial additional work in traditional networks. Moreover, legacy networks suffered from limited flexibility and lacked the capability for immediate, fast, and productive responses to network traffic demands. The main advantage of Software-Defined Networks lies in the centralized control and the separation of the data plane (transmission) and the control planes. The control plane ensures the management of programmable network devices, while the responsibility of forwarding packets according to the processing forwarding tables received from the controller lies solely with the switches. In recent years, SDN has witnessed widespread adoption, not only in large corporate and institutional networks but also in home networks. This is primarily due to the cost-saving opportunities provided by it, particularly in the energy domain, where costs have significantly surged. When examining network expenses, it becomes apparent that a considerable proportion is attributed to energy consumption. Consequently, numerous investigations have been carried out over the past few years and decades to reduce these expenses.

Several approaches exist for saving energy in SDN networks. They operate either on a hardware basis or with software solutions. From a physical point of view, energy savings are derived from optimizing the memory utilization of the switches. Managing the Ternary Content Addressable Memory (TCAM) [3] offers practical solutions. It is crucial to compress the contents and, if necessary, increase memory capacity to contribute to energy savings. However, it is important to note that memory capacity cannot be expanded infinitely.

Software-based energy savings can be accomplished through the controller by leveraging central control. In this scenario, different algorithms can be utilized to influence the behavior of the network ensuring that energy savings do not come at the expense of network performance. SDN energy efficiency methods can primarily be categorized into four groups: Compression TCAM [3], End Host-Aware [4], Traffic-Aware [5], and Rule Placement [6].

This article introduces a novel algorithm called the Modified Heuristic Algorithm for Energy Saving (MHAES), which undergoes a comparative evaluation with eight commonly employed methods across various topologies to assess its energy efficiency. The results presented below stem from an investigation that primarily focused on three main categories of energy-saving methods: compressive TCAM, traffic monitoring, and the rule placement method. Although the endpoint-aware method was also examined, it was not considered relevant to our research as our primary focus revolved around campus level or smaller networks. Within the three selected categories, the options that greatly facilitated the operation of the heuristic algorithm being proposed in this article were thoroughly analyzed. Specifically, the Rectilinear [7] and CAM Razor [8] methods were examined for Compression TCAM, both prioritizing either rule or content compression. From the research perspective, both methods held significance, as the goal was to convey to the switches as many basic rules as possible while minimizing energy consumption. Traffic-aware options such as Green Abstraction Layer (GAL) [9], Exclusive Routing (EXR) [10], Strategic Greedy Heuristic (SGH) [11], or Routing for Minimization of Active Devices (RMAD, RMAD+) [12] were also analyzed. They proposed efficient solutions for the current investigation, enabling us to achieve energy savings with the implemented modifications. Additionally, rule placement methods such as Energy-Aware Routing (EAR) [13] and DevoFlow [14] were also considered and played a vital role in the research. These methods were modified resulting in the proposed heuristic algorithm. The primary objective of this paper is to present this algorithm and the achieved outcomes.

The rest of this paper is as follows. First, Software-Defined Networks (SDN) are introduced briefly in Section 2. Then, energy-saving solutions in SDNs are reviewed in Section 3. Section 4 presents the proposed new algorithm, while the simulation results are described in Section 5. Finally, the conclusions are drawn in Section 6.

## 2. Software Defined Networks

**Figure 1:**
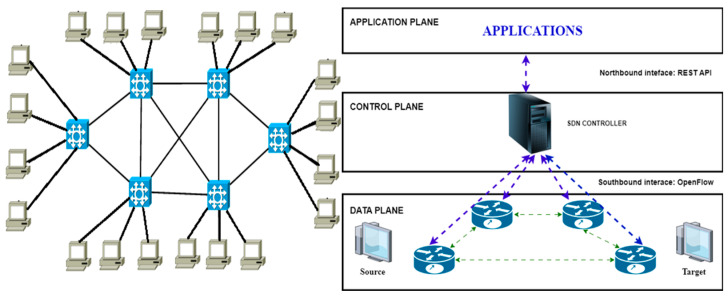
Traditional vs. SDN networks.

**Figure 2:**
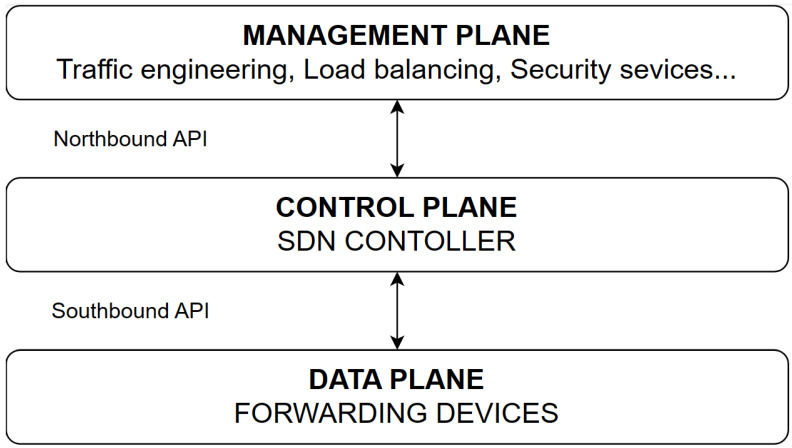
SDN architecture.

SDNs [15,16] completely separate controlling from the data transmission in contrast to the solution used in traditional networks. All decisions in the traditional network depend on the routers (e.g., packet forwarding), while in SDNs, the controller decides the best way for the packets using the centralization (Figure 1). Three main layers are distinguished in the SDN architecture (Figure 2), i.e., the data plane [17] (infrastructure layer), the control plane [18] and the application plane [19].

Data plane: The data layer houses the network infrastructure, which consists of data transmission devices commonly referred to as switches. These devices ensure the delivery of packets from the source to the destination based on instructions received from the controller. In traditional networks, routers continuously exchange messages to determine the correct route. In SDN networks, switches inform the controller about the current topology status, enabling decision-making on appropriate traffic control in the upper layer. Data transmission information is then relayed to the relevant network devices through the southbound interface. Error-free communication with the control layer requires the use of the appropriate protocol, such as the open standard OpenFlow [20,21]. This protocol provides a set of commands to modify the state of devices participating in an SDN.

Control Plane: The control layer encompasses the SDN controller(s) responsible for centralization in SDN. Controllers make decisions regarding rule placement and instructions for traffic control. However, centralization can pose challenges, including network security, scalability, and energy management, especially when controllers become overloaded. It is important to note that an SDN controller does not necessarily solve these tasks in larger networks. Attention should be given to the location of SDN controllers, as distributed control, error management, and energy consumption pose additional challenges. The network hypervisor and network operating system, located in this layer, aid in resolving these functions.

The control layer’s third component is the northbound interface, facilitating communication between the controller and applications in the management layer. The northbound interface commonly employs open-source application programming interfaces (APIs) that can be easily utilized and modified according to specific requirements.

Application plane: The application layer comprises SDN network applications and programming languages. This layer provides a user interface to configure desired settings, including load distribution among potential routes, network security settings, rule system creation, and energy consumption modifications.

By employing SDN applications and proper monitoring, rapid responses to network changes can be achieved, such as addressing load balancing issues, lost routes leading to topological changes, or unexpected attacks. Centralization eliminates the need for local reconfiguration, allowing for quick and efficient changes through the controller [22]. This capability is beneficial for network operation from all perspectives, and our article focuses on potential solutions for improving energy usage.

## 3. Energy Saving Solutions in SDN Networks

With the emergence of the energy crisis, the task of reducing energy consumption through centralization is becoming increasingly challenging for SDN networks as well. Moreover, accurately monitoring and tracking energy consumption is also crucial. Various techniques can be employed in SDN networks to achieve energy saving. It should be noted that in these networks, the decisions are made by the controller. SDN switches can be configured to operate in an active or sleeping state, bandwidth can be limited, and rules for network operation can be defined and sent to the respective devices. This section presents an overview of these different energy saving methods.

### 3.1. Compression TCAM Solution

Switches utilize a specialized memory known as Content Addressable Memory (CAM) [23] to store and retrieve data. Unlike regular RAM, which relies on memory addresses, CAM allows direct content querying without the need for specifying addresses. CAM offers faster access compared to RAM and its tables yield either a true (0) or false (1) result. This type of memory is particularly useful for creating tables that require exact matches, such as MAC address tables. However, CAM’s limitation lies in its ability to only check for complete matches of ones and zeros. To address this limitation, the solution lies in Ternary Content Addressable Memory (TCAM) [24,25].

TCAM serves as a specialized form of CAM, capable of storing and retrieving data based on three different input parameters: 0, 1, and X (“do not care”). The inclusion of the “do not care” input parameter, acting as a wildcard, enables TCAM to perform more detailed searches as intended. TCAM proves valuable in finding longest matches and also stores additional information such as Access Control Lists (ACLs) [26] and other higher-level task-related data. While TCAM functions well in SDN switches for efficient routing, its drawbacks include high cost, significant power consumption, and excessive heat generation. These challenges pose a considerable obstacle for central controllers and other network devices. However, it is worth noting that the use of TCAM ensures that the application of ACLs does not impact the performance of the switch. Multiple TCAMs can be present in a switch, enabling the simultaneous or parallel evaluation of incoming and outgoing security ACLs, offering a substantial energy-saving advantage.

Considering the expense and energy consumption associated with TCAM usage, finding a solution to address these issues becomes imperative. One possible method is the compression of TCAM. Two potential compression approaches for TCAM are rule compression and content compression.

In SDN, switches make their decisions based on the forwarding rule identified in TCAM. Each entry in the process table defines a matching rule, specifying a predefined operation for each case. Upon packet arrival, the forwarding device identifies the highest priority matching rule and executes the requested action. Various solutions have been developed to compress these rules, ensuring a more efficient utilization of TCAM.

The Rectilinear [7] approach leverages the features provided by SDN, such as centrally programmable interfaces and the dynamic definition of actions for forwarding specific packets (content compression). In the case of compression, the bit size is reduced to store only vital information necessary for packet routing. Each flow is assigned a unique flow ID to ensure proper identification. Forwarding switches modify the packet headers to incorporate the flow ID, facilitating packet classification by switches. SDN switches employ a shorter label representation compared to the original bit number to identify flows, thereby conserving energy.

The rule compression method, known as the CAM Razor [8] method, defines a four-step process. In the first step, a reduced decision diagram is created for the given rules. The second step involves considering rules associated with non-central nodes and minimizing them using dynamic programming techniques. Subsequently, rules are generated from the decision diagram in the third step. In the fourth and final step, unnecessary or redundant rules are eliminated from the process. 

Compact TCAM [8,24,25] is a solution that reduces the size of TCAM process entries and uses shorter tags for process identification. This can be used to reduce the size of forwarding rules, so the TCAM space can be optimized. For the efficient use of TCAM, a scheme was created, which can be used to compress forwarding rule in overlapping switching nodes, thus reducing the occupied area in TCAM.

### 3.2. End Host-Aware Solution

The utilization of End Host-Aware solutions involves the common practice of shutting down less utilized servers and redirecting their tasks to other servers. This approach proves particularly advantageous for energy conservation in data centers.

ECODANE [27] serves as a valuable solution for energy saving in data centers. The implemented method consists of five logical modules: the data center network, optimizer, power control, forwarding module, and traffic generator. To create a data center network, the project creators utilized the Elastic-Tree network [28] and simulated it using Mininet [29]. The task of the optimization module is to determine the minimum energy network segment, requiring the fewest switches and links, while maintaining traffic conditions corresponding to the requests and upholding quality of service (QoS) standards. A topology-aware heuristic [30] was developed to assist the optimizer in quickly determining the suitable subset for the power controller and forwarding module. 

Leveraging OpenFlow, the power control module can modify the power supply status of ports or entire switches (i.e., turning them off, on, or enabling energy-saving mode) using the options provided by the Python API of Mininet. The function of the forwarding module is to optimize routes within the data center, employing a hierarchical load balancing routing algorithm [31]. Although the optimizer and forwarding module are implemented separately, they closely collaborate. The forwarding module operates based on the data received from the optimizer, but in the absence of information, it sets all non-active network devices to the “on” state. The traffic generator contributes to the simulation process.

It is important to note that our research does not specifically focus on data center networks. Instead, we concentrate on optimizing energy consumption in institutional, local, and large company networks within the region. Therefore, this method did not play a prominent role in our study, and we did not compare it to our own algorithm.

### 3.3. Traffic-Aware Solution 

The energy efficiency solution, focusing on traffic awareness, highlights the fact that network switches are not always fully utilized. By optimizing the on and off state of these devices, energy savings of up to 40% can be achieved. The key idea is to switch on these transmission devices only when necessary and switch them off otherwise, thereby conserving energy [32]. While energy saving is crucial, it is equally important to consider the quality of service (QoS) [33] since a significant reduction in QoS can have a more detrimental impact on our optimized network than the energy saved.

The Green Abstraction Layer (GAL) [9,10] method represents a traffic-aware solution that capitalizes on the communication capabilities between network devices, with the querying of this communication playing a pivotal role in energy saving. The GAL abstraction encompasses two main models: the Energy-Aware State (EAS) [12] and the Energy Optimizer module. EAS defines logically independent units of the network, such as switches or traffic management operations. Data collected from these units are transmitted to the controller, which, considering the actual requirements, applies the most effective strategies across the network devices. The Energy Optimizer module calculates the optimal state to minimize energy consumption based on information received from EAS. The GAL operation consists of three distinct stages: discovery, provisioning, and monitoring. In the discovery phase, information is collected, and network devices inform the controller about their energy usage. This process can be time-consuming and energy-intensive due to the varying number of devices depending on the network size. The provisioning stage, on the other hand, is faster and more efficient as it leverages basic settings and rules whenever possible to set the network nodes to their optimal energy states. Following the provisioning stage, the monitoring phase ensures continuous optimal network operation. The controller receives real-time information about the current state of each entity through monitoring, and if necessary for network operation, the triple process restarts.

Exclusive Routing (EXR) [30] represents a method to enhance fair-sharing routing (FSR) [34], a general routing approach for efficient distribution. FSR evenly distributes flows over a subset of links, ensuring fairness without delay. However, this method operates with reduced capacity, failing to utilize the full capacity available. By implementing EXR, energy can be saved by utilizing the full capacity of active links and putting additional switches to sleep. The primary aspect of this solution lies in eliminating low link and switch utilization. If all routes are used, but a route has temporarily suspended flows with lower priority than the current flow, the flow becomes active, and the package is forwarded via a lower priority route. Otherwise, the flow remains suspended. If multiple options are available, the switch with the fewest idle states is selected. Upon the successful completion of a process, the controller resets the necessary flows.

Tests conducted using the OpenFlow protocol have revealed that EXR enables energy savings regardless of whether the network is idle or busy, achieving effective utilization through connection structure application or capacity utilization modifications. Prioritizing flows can enhance the network’s flexibility in utilization, but careful attention must be paid to avoid delays. It is essential to note that network flexibility in this solution assumes the presence of backup routes and swift response, as failing to meet these conditions may result in an unacceptable level of service.

The Strategic Greedy Heuristic (SGH) [11] algorithm serves as an effective means to optimize the energy consumption of Software-Defined Networks (SDNs). In SGH, participating access nodes in a flow share their load information with other nodes, and the controller optimizes traffic flow accordingly, putting idle devices to sleep whenever possible. SGH considers three aspects: capacity limitation to ensure efficient connections without underutilization or overload [35,36,37], assigning flows to paths with adequate capacity for each traffic demand, and shutting down nodes where necessary.

Routing for Minimization of Active Devices (RMAD, RMAD+) [12] presents a highly beneficial energy-saving solution for SDN networks. This method takes into account the energy savings achievable by turning devices on and off. The outlined method primarily utilizes the Open Shortest Path First (OSPF) [38] protocol for route determination, which proved to be an effective and swift solution. RMAD identifies the most active links using the shortest path, extending the sleeping time of already inactive units to achieve greater energy savings. The improved RMAD+ further limits the number of hops on a given path and selects the path with the fewest active nodes. It is important to note that the selection of a path with fewer active nodes does not necessarily equate to the shortest path determination.

### 3.4. Rule Placement Solution

The Rule Placement method [6,13,39] necessitates the placement of information in switches to ensure proper packet forwarding from the source to the destination address. The crucial aspect of these techniques lies in the reception of accurate, fast, and easy-to-implement rules by the processing devices. The ideal solution is centralized control, where the controller provides the most optimal transmission paths. The downloading of rules to the switches is a significant aspect of this technology. Although heuristic-based algorithms are not foolproof, they contribute to finding the optimal solution for network enhancement. It should be noted that switches have finite memory capacity, but this can be optimized by defining basic traffic control rules and preloading them onto the switches.

The Energy-Aware Routing (EAR) [13] solution assumes an infinite number of rule spaces, which is not accurate due to the limited size of the TCAM, as mentioned previously. Giroire et al. [13] developed a method to overcome the rule space limitations in energy-aware routing. The initial step involved creating a default rule, which is selected when no predefined rule is available for packet forwarding, indicating that the forwarding device lacks the necessary information to determine the possible route. Deploying these default rules to devices is time-consuming and energy-intensive, but it can yield long-term benefits if properly implemented. A crucial aspect is that these basic rules are only sent to one default port from the controller, resulting in energy savings throughout the network. The developers of the method considered the determination of the default port as a key element of their solution. It is important to note that each switch has only one default port. To address this, a heuristic algorithm was created, aiming to find the best possible forwarding route for received requests while considering storage capacity and rule limitations. The routing table is continuously populated with appropriate routes until the TCAM reaches its capacity. The default port is determined based on the port involved in the most management tasks. Taking this into account, the number of active links is reduced, switches with lower loads are put into sleep mode, and their responsibilities are assumed by other active devices, resulting in energy savings. This solution not only conserves energy but also reduces the rule area size, decreases delay time, and minimizes communication with the controller, which is crucial in a corporate environment.

Another viable and beneficial solution is DevoFlow [40], which essentially modifies an OpenFlow controller. The solution involves breaking the connection with the controller to offload certain tasks to the switches, thereby reducing the workload on the controller and leading to significant energy savings. The controller continuously gathers network information, consuming substantial energy and hindering the fast transmission of large packets. To expedite switch control, rule cloning was introduced. When a flow matches another flow, the switch clones the rule, reducing the usage of TCAM and minimizing communication with the controller. The authors also proposed a local mechanism, enabling switches to make decisions without consulting the controller for certain packet transmissions, leveraging the use of basic rules.

In terms of the research (Table 1 shows the examined solutions), the Traffic-Aware solution, combined with rule placement options (and the utilization of Compression TCAM), proved to be highly beneficial. The reason is the Traffic-Aware and the Rule Placement solutions have a very useful relationship. Important feature of Traffic-Aware is elasticity and topology awareness. Talking about the Rule Placement solution, the knowledge of the entire topology is closely related to these two properties, which is essential for choosing the best route. Knowing the full network topology means having a global view. It is the ability to decide which rules have to be placed on which switch, which depends on the total network information, end-point policies, and routing policies. The set of these rules is the rule space. Rule spaces can be reduced by reducing the number of active switches and links. It is important to note that attention must be paid to which rule is defined for proactive operation (before the packet arrives) and which is for reactive operation (reaction to new packets).

The insights gained from all these methods played a pivotal role in shaping the development of the Modified Heuristic Algorithm for Energy Saving (MHAES), as detailed in the previous section (refer to Table 1 for the analyzed solutions). Notably, the Traffic-Aware solution, coupled with strategic rule placement and the adoption of Compression TCAM, demonstrated significant advantages. These methodologies collectively influenced the refinement and formulation of the Modified Heuristic Algorithm for Energy Saving (MHAES), which will be elaborated upon in the following section.

## 4. Energy-Aware Approach—Modified Heuristic Algorithm for Energy Saving (MHAES)

In the preceding section, several methods were presented that can effectively contribute to energy savings in SDNs. The mathematical models of the Traffic-Aware solution and the Rule Placement solution [41,42,43,44,45], which will also be shown in this section, had a significant impact on the design of the new algorithm presented here. Moreover, the mathematical background of the proposed algorithm will be detailed as well, along with the algorithm itself.

### 4.1. Mathematical Model for Traffic-Aware Solutions 

Network traffic, which refers to the number of packets transmitted on the network at a given time, can be managed to avoid congestion, promote proper load distribution, and save energy. For optimal route selection, the status information of the devices used in the network is periodically collected by the SDN controller. This data assist in determining the network rule system, enabling quick reactions in case of possible failures. The Traffic-Aware model [12,32,35,36,37], which is a solution aimed at minimizing power consumption by deciding when each device should be put to sleep under low traffic conditions, can be utilized through SDN-centralized management. Table 2 shows the list of notations used.

In terms of energy consumption, links and switches are the main participants in SDN networks. The reduction in the number of switches and connections required for network traffic transfer is crucial for achieving more favorable energy savings.

Network traffic can be illustrated by an undirected weighted graph *G = <Z, E>.* Here *Z* and *E* represent the number of switches and connections, where *z_i_ ∈ Z* and *e_i_j ∈ E*. The connection, or link, between switches *z_i_* and *z_j_* is denoted by *e_ij_*. The current state of the switch is determined by the following equation.

(1) $$S_{i}=\left\{\begin{array}{c} 1\;\mathrm{if}\;\mathrm{switch}\;z_{i}\;\mathrm{is}\;\mathrm{active}\\ 0\;\mathrm{otherwise} \end{array}\right\}$$

Equation (2) aims to minimize the total energy consumption of the network through the utilization of switches and connections. The energy consumption of the links and switches is represented by the first and second terms, respectively.

The energy consumption of the connection *e_ij_* and the switch *z_i_* is denoted as *PC_ij_* and *PCS_i_,* respectively. The network traffic flow, represented by *f*, belongs to the set *F*, where *f* can be defined as *f = (s_r_ d_s_ f_r_).* The flow rate is denoted as *f_r_*, while *s_r_* and *d_s_*, both belonging to the set *Z*, represent the source and destination switches, respectively,

(2) $$Minimalize\left(\sum_{\forall f}\sum_{\forall e_{ij}}F_{ij}\times {PC}_{ij}+\sum_{\forall S_{i}}S_{i}\times {PCS}_{i}\right)$$

subject to:

(3) $$\sum_{\forall f}F_{ij}\times \leq {BW}_{ij},{\forall e}_{ij}$$

(4) $$\sum_{\forall f}F_{ai}=\sum_{\forall f}F_{ib,}z_{i}\neq s_{r},d_{s}\in f_{s_{r},d_{s},\lambda_{f}}$$

(5) $$F_{mj}=F_{in},z_{m}=s_{r},z_{n}=d_{s},{\forall e}_{mj},{\forall e}_{in}$$

(6) $$F_{ij}\leq S_{j},{\forall z}_{j}\in z$$

(7) $$F_{ij}\leq S_{j},{\forall z}_{i}\in z$$

(8) $$S_{i}\leq \sum_{\forall f}\left[f_{ij}+f_{ji}\right],{\forall z}_{i}\in z$$

The flow rate between the switches is specified by Equation (3), which ensures that it does not exceed the maximum capacity of the available connection. The inflow and outflow values are given by Equation (4). Equation (5) governs the flow from the source to the destination. Equations (6) and (7) ensure that the flow cannot be directed towards any sleeping switches. Assistance in deactivating idle switches is provided by Equation (8).

### 4.2. Mathematical Model for Rule Placement Solutions 

Another energy saving method, as already mentioned, is Rule Placement solution [6,13,39,41,42,43,44,45]. An important aspect of this method is TCAM, in which significant energy savings can be achieved by controlling the number of flow rules. TCAM memory is finite, and an important aspect is that this memory is also expensive. The energy consumption of the SDN network can be influenced by the number of entries stored in the TCAM. By using the rule placement method, the number of rules placed in the TCAM can be influenced, for example with the default rules, or possibly with other replacement methods.

The goal of this solution is to minimize flow inputs, thus increasing energy efficiency. To represent the network traffic, we use an undirected weighted graph *G = <Z, E>* where *Z* and *E* represent the number of switches and links, such that *z_i_ ∈ Z* and *e_ij_ ∈ E*. The e_ij_ is a connection between switches *z_i_* and *z_j_*. The bandwidth of the given link is presented by *w_ij_*. The state of the switch is given by the following Equation (9).

(9) $$S_{i}=\left\{\begin{array}{c} 1,\;\mathrm{if}\;\mathrm{switch}\;z_{i}\;\mathrm{is}\;\mathrm{active}\\ 0,\;\mathrm{otherwise} \end{array}\right\}$$

Considering the free capacity of resources, the matrix of rules can be described as follows (10). Rules represent flows associated with switches:

(10) $$a_{if}=\left\{\begin{array}{c} 1\;\mathrm{if}\;\mathrm{rule}\;\mathrm{representig}\;\mathrm{flow}\;f\;\mathrm{is}\;\mathrm{installed}\;\mathrm{on}\;\mathrm{switch}\;z_{i}\\ 0\;\mathrm{otherwise} \end{array}\right\}$$

The size of the matrix is determined by the number of switches in the matrix. The edge is *e_ij_*, and whether it is asleep or not is decided by the binary variable *L_ij_*.

(11) $$L_{ij}=\left\{\begin{array}{c} 1,\;\mathrm{if}\;\mathrm{edge}\;e_{ij}\;\mathrm{is}\;\mathrm{active}\\ 0,\;\mathrm{otherwise} \end{array}\right\}$$

The status of the flow through the edge *e_ij_* is refreshed by the variable *K_ij_*, which gives back a binary result.

(12) $$K_{ij}=\left\{\begin{array}{c} 1,\;\sum_{f\in F}F_{ij}\geq 1\\ 0,\;\mathrm{otherwise} \end{array}\right\}$$

The number of rules present in switches is given by Equation (13). The capacity of the connection fixedly determines the number of flows that can pass through the given link, and this can be seen in Equation (14). Of course, the number of rules built into the switch cannot exceed the switch capacity, Equation (15). Equation (16) shows the state of the flow passing through edge *e_ij_*. Equation (17) puts the unused connection to sleep. If this reference is used in any process, it must become active. It can be seen in Equation (18). Equation (19) contains the rule of flow retention.

(13) $$Minimalize\sum a_{if}\forall f$$

is subject to:

(14) $$\sum_{\forall f}F_{ij}\times \lambda_{f}\leq W_{ij},{\forall e}_{ij}$$

(15) $$\sum_{\forall f}F_{ij}=G_{i},{\forall z}_{i}$$

(16) $$F_{mj}=F_{in},z_{m}=s_{r},z_{n}=d_{s},{\forall e}_{mj},{\forall e}_{in}$$

(17) $$F_{ij}\leq S_{j},{\forall z}_{j}\in z$$

(18) $$F_{ij}\leq S_{j},{\forall z}_{i}\in z$$

(19) $$S_{i}\leq \sum_{\forall f}\left[f_{ij}+f_{ji}\right],{\forall z}_{i}\in z$$

### 4.3. Mathematical Model and Algorithm of MHAES

As the starting point of the development of the Modified Heuristic Algorithm for Energy Saving, an analysis was carried out to determine the components upon which the energy consumption of switches depends. For this purpose, the following formula was utilized.

*E_switch_ = E_basic_ + E_configuration_ + E_openflow_ + E_control,_* (20)

where *E_basic_* denotes the basic performance of the switch, *E_configuration_* is configuration speed, *E_openflow_* refers to the energy consumption of the processed OpenFlow traffic (operations), while *E_control_* pertains to the energy consumption of the control traffic (which depends on the speed of outgoing and incoming packets).

During the investigation, focus was placed on determining which components’ energy consumption could be influenced to achieve greater energy saving. *E_basic_* and *E_openflow_* offered limited opportunities for reducing energy usage but modifying the variables *E_configuration_* and *E_control_* showed promise in solving the problem.

In this investigation an undirected weighted graph *G = <K, E>* was used to represent the network traffic. Here, *K* includes both hosts and SDN switches (*K = H U Z*), and *E* represents the number of connections, such that *z_i_ ∈ Z* and *e_ij_ ∈ E*. The connection between switches *z_i_* and *z_j_* is denoted by *e_ij_*. The basic rule *B* applied between hosts was taken into account. For each *R, H_r_* represents the default traffic control rule set.

The premise underlying the approach was to minimize communication between transmission devices and the controller, thereby achieving significant savings in delay time, capacity utilization, and energy consumption. To enable reduced communication, an algorithm was conceived that detects active and sleeping SDN-compatible switches and seeks to avoid activating switches already in a sleeping state. Whenever possible, as many default and local rules as possible were applied to determine the proper forwarding of packets between the source and destination addresses without relying on central communication. The primary goal was to minimize energy consumption in the SDN network.

When developing the mathematical model, the well-established solutions developed in the aforementioned Traffic-Aware and Rule Placement approaches were taken into account, supplemented by the defined expectations aimed at reducing network energy consumption. Table 3 shows the designations of basic and special input data, while Table 4 contains the decision parameters.

The total energy consumption of SDN switches participating in the network is minimized by the task of function (21). Flow constraints are contained in Equation (22). Equation (23) indicates that the traffic forwarded on each connection’s link is either less than or equal to the available capacity. Constraint (24) ensures that if a connection is in a sleep state, *CA_e_* = 0, indicating it has no capacity. However, if the link is active, its capacity is limited by *CA*, which represents the maximum capacity of the link.

Constraint (25) guarantees that when a switch *z* is in the off state, the capacity of both the incoming and outgoing links must be 0, i.e., *CA_e_* = 0. Equation (26) ensures that if a request *r* does not impact the link e, the traffic associated with the request will be 0 (*Y_re_* = 0). Equation (27) determines the number of processes belonging to the OpenFlow protocol that should be set on the *z* switch.

(21) $$X=\min{(E}_{basic}\sum_{z}y_{z}+E_{configuration\_po}\sum_{e}z_{e}+E_{configuration\_sp}\sum_{e}{CA}_{e}+E_{openlow}\sum_{z}r_{z})$$

is subject to:

(22) $$\sum_{e\in k^{out}\left(z\right)}Y_{re}-\sum_{e\in k^{in}\left(z\right)}Y_{re}=\left\{\begin{array}{c} h_{r},\;if\;z=s\left(r\right);\\ -h_{r},\;if\;z=t(r)\\ 0,\;otherwise \end{array}\right\},\forall r\in R,\forall z\in Z\;\;$$

(23) $$\sum_{r}Y_{re}\leq {CA}_{e}\forall e\in E$$

(24) $${CA}_{e}\leq {CA}_{Z_{e}},\;\forall e\in E$$

(25) $$\sum_{e\in k^{out}(z)}{CA}_{e}-\sum_{e\in k^{in}(z)}{CA}_{e}\leq {BN}_{Y_{z}}\forall z\in Z\;$$

(26) $$Y_{re}\leq {BNy}_{re},\forall r\in R,\forall e\in E$$

(27) $$\sum_{e\in k^{out}\left(z\right)}\sum_{r}y_{re}=r_{z},\forall z\in Z$$

The modified heuristic algorithm (Algorithm 1) has been developed using the above-presented model.
**Algorithm 1** The modified heuristic algorithm**FOR EACH** CuRe **IN** R **DO**  **IF** CuRe is first request **THEN**    CuPa = Shortest_Path(CuRe)    CPo = Power_Value(CuPa, CuRe)    Update(CuPa, ASW, SSW, AL, CA, F)  **ELSE**    P = All_Available_Paths(CuRe)    SP = []    **FOR EACH** CuPa **IN** P **DO**      **IF** Available_Capacity(CuPa)>TCA **THEN**        CPo = Power_Value(CuPa, CuRe)        SP ← CuPa, CPo      **END IF**    **END FOR**    OP = Minimum_Power_Path(SP)    Update(OP, ASW, SSW, AL, CA, F)  **END IF** **END FOR**

In the algorithm, *R* represents the list of traffic requests, and *CuRe* represents the current request that needs to be examined. *TCA* is the threshold value of the link capacity defined manually [46]. *ASW* represents the list of active SDN switches, while *SSW* contains the set of switches in a sleeping state. *AL* is the list of active links, while *CA* represents the capacity of the link. *F* represents the active flows in each SDN switch. *CPo* denotes the power consumption of the current request, and *SP* includes the list of selected paths and their corresponding *CPo*.

The main goal was to determine a route that would minimize the energy consumption of the network. The algorithm treats the first request separately from subsequent requests. For the first incoming request, the algorithm considers it as the shortest and most efficient path in terms of energy consumption and updates the required values accordingly. In the case of requests following the first request, the first task is to define the set of all available routes that can be assigned to the given request. Next, the method selects the set of paths with a capacity greater than the specified capacity threshold (20%) and determines their energy consumption. Next, it chooses the path that results in the smallest energy increase in the network. After determining the path with the minimum value, the necessary values are updated accordingly.

In the *Power_Value* function, as depicted in Algorithm 2, the algorithm determines whether certain actions need to be taken based on the threshold number of active switches. These actions include activating a new switch if necessary, modifying link settings (e.g., increasing speed with *Active_Links_Update* (*CL*, *CuRe*)), or adding a new flow entry to the existing ones as requested (*Basic_Flow_Update* (*CuRe*)).

A switch in a sleeping state can only be awakened under the condition that the switch intended for use by the link is not active, meaning there is no active connection between the source and target, and the number of active SDN switches does not exceed the specified threshold number.
**Algorithm 2** Function Power_Value**Power_Value(path, CuRe)** **FOR** all CL **IN** path **DO**  Active_Links_Update(CL, CuRe)  Basic_Flow_Update(CuRe)  **IF** origin(CL) or destination(CL) not in ASW **THEN**    **IF** count(ASW)>TASW **THEN**      **RETURN** infinite    **END IF**    Active_Switches_update(ASW, SSW)  **END IF**  power=Calculate_Power(CL, CuRe) **END FOR** **RETURN** power

## 5. Simulation and Results

The algorithm was tested in Net2Plan [47,48,49] using several different environments. For this purpose, two network topologies, i.e., Simmons and eon, built into the software, were employed with different numbers of nodes. The tests were conducted on fixed wired networks with 15, 30, 45, and 60 nodes. In each case, six different solutions were compared to the newly created algorithm five times starting from different nodes. The power consumption was analyzed when all switches and links were active. Additionally, the Rectilinear, DevoFlow, EXR, RMAD, RMAD+, and Giroire et al.’s heuristic algorithm solutions were tested and compared against the new MHAES solution.

**Figure 3:**
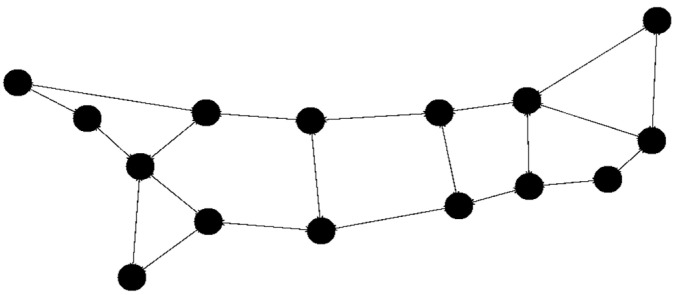
Network topology with 15 nodes (changed topology).

**Figure 4:**
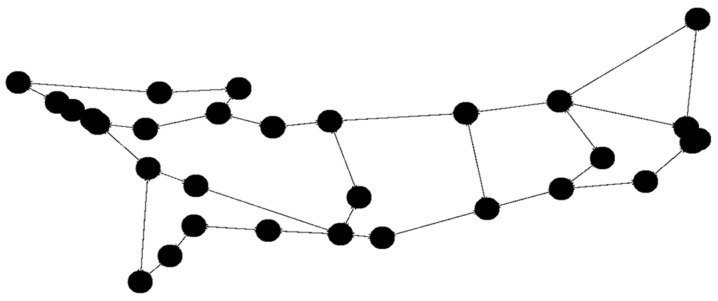
Network topology with 30 nodes.

**Figure 5:**
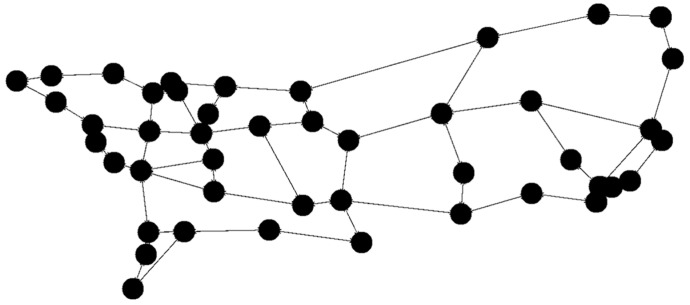
Network topology with 45 nodes (changed topology).

**Figure 6:**
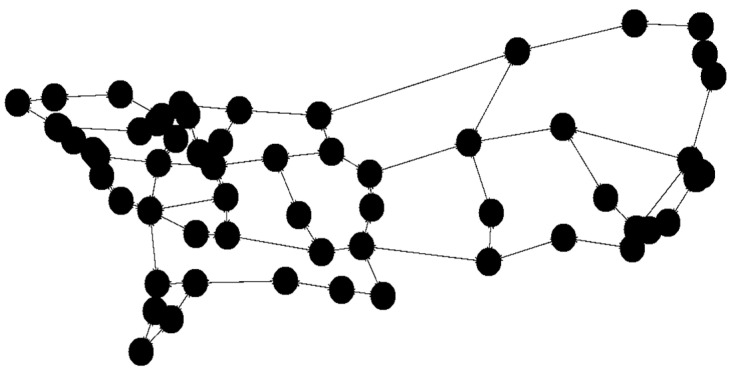
Network topology with 60 nodes.

The initial tests utilized the built-in networks Simmons_N30_E72 and Simmons_N60_E154 E154. To broaden the scope of testing, selected solutions were also tested with 15 nodes. For the 15-node solution (Figure 3), the network topology Simmons_N30_E72 was modified by removing several links and nodes (resulting in 43 links). With 30 nodes we use the original Simmons_N30_E72 (with 72 links) topology (Figure 4.). The topology with 45 nodes (Figure 5) was created by deleting nodes and links from the Simmons_N60_E154 network (resulting in 122 links). In Figure 6. can see the original topology Simmons_N60_E154 (with 154 links).

**Figure 7:**
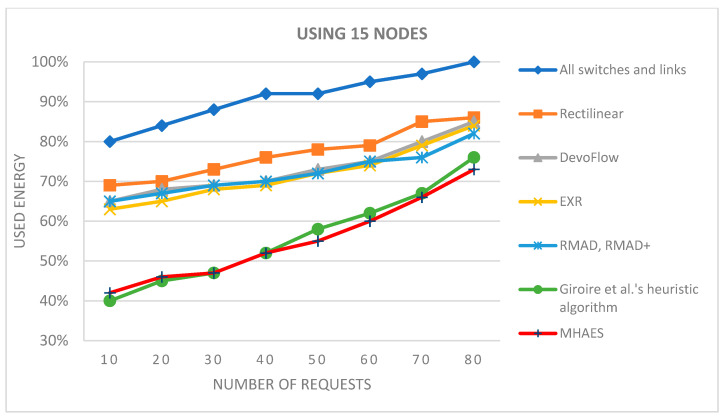
The average energy consumption with 15 nodes depending on the number of requests.

**Figure 8:**
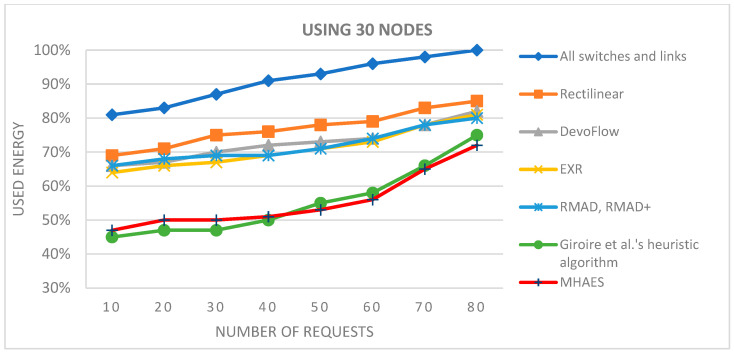
The average energy consumption with 30 nodes depending on the number of requests.

**Figure 9:**
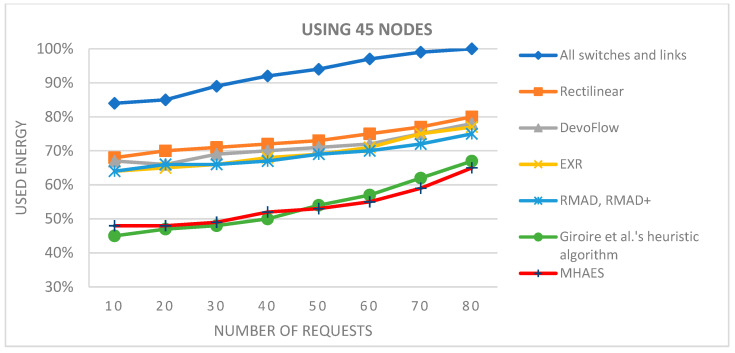
The average energy consumption with 45 nodes depending on the number of requests.

**Figure 10:**
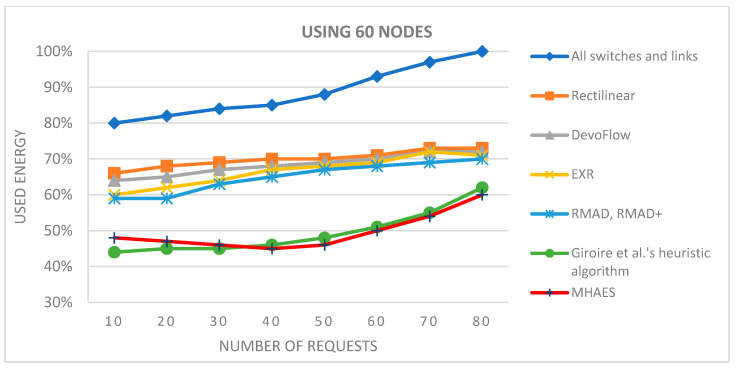
The average energy consumption with 60 nodes depending on the number of requests.

The simulation average results are depicted in the diagrams presented in Figure 7, Figure 8, Figure 9 and Figure 10, representing networks with 15, 30, 45, and 60 nodes, respectively. The y-axis indicates the energy consumption relative to the expected amount, where 100% corresponds to the scenario with all links and nodes active. All tested methods consistently consumed less energy compared to this scenario. In the case of 15 nodes (Figure 7), the Rectilinear, DevoFlow, EXR, RMAD, and RMAD+ solutions exhibited energy savings of average 10–15% for a small number of requests and up to 15–20% for a larger number of requests on average. Giroire et al.’s heuristic algorithm and the MHAES consistently outperformed the other methods, achieving energy savings of around 25% for 80 requests. The MHAES demonstrated the best performance, surpassing Giroire et al.’s heuristic algorithm by an additional 2% in energy savings on average. This was mainly due to the fact that more information reaches the nodes in the initial stage, which, although it requires more energy in the case of continuous increases in requests, due to less frequent contact with the controller, it uses its own local information and can save energy. It is important that the capacity and the number of sleeping nodes decisively influence the amount of energy savings. In this case, these numbers are constant values given through experience, which will be determined in the future, taking into account the actual needs. Notably, this study served as a starting point and focused on network topologies typically found in smaller companies.

Moving on to networks with 30 nodes (Figure 8), the initial average energy savings with the MHAES and Giroire et al.’s heuristic algorithm were slightly lower compared to the 15-node scenario. However, they still outperformed the other examined methods, demonstrating only minimal loss. The MHAES began performing noticeably better after 25 requests, achieving significant energy savings. Similar energy savings to the 15-node test were attained for 80 requests in this network.

Figure 9 and Figure 10 display the results for networks with 45 and 60 nodes, respectively. Once again, the MHAES and Giroire et al.’s heuristic algorithm outperformed the other solutions. The Rectilinear, DevoFlow, EXR, and RMAD methods achieved average energy savings of 20–25% for almost 80 requests in the case of 45 nodes and 25–30% for 60 nodes. However, the MHAES and Giroire et al.’s heuristic algorithm, after the initial rule placement load, consistently achieved substantial energy savings compared to the aforementioned methods. With 40–45 requests, the MHAES consistently outperformed its competitors, achieving nearly 40% energy savings compared to the scenario where all switches and links are active. In the network with 60 nodes, the MHAES maintained a 2% average energy savings advantage over Giroire et al.’s heuristic algorithm after 45 requests, which persisted even after 80 requests. It is worth noting that due to the larger number of links, all tested methods exhibited smaller savings compared to the first series of tests. The deviation from the average value in the negative direction was not greater than 1.2% in all cases, while the maximum value in the positive direction was 2.5%. The network performance performed similarly in all cases examined, and no significant differences were observed.

**Figure 11:**
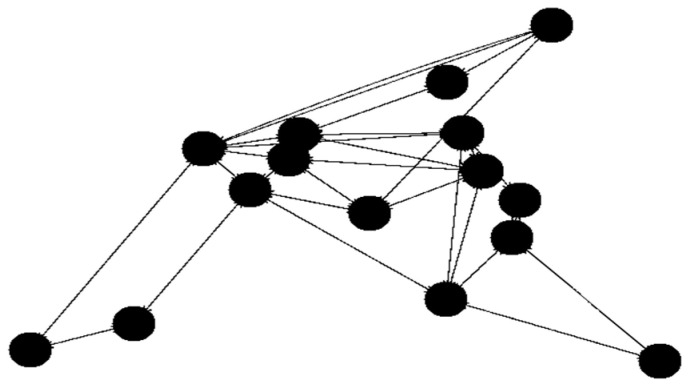
Network topology with 15 nodes.

**Figure 12:**
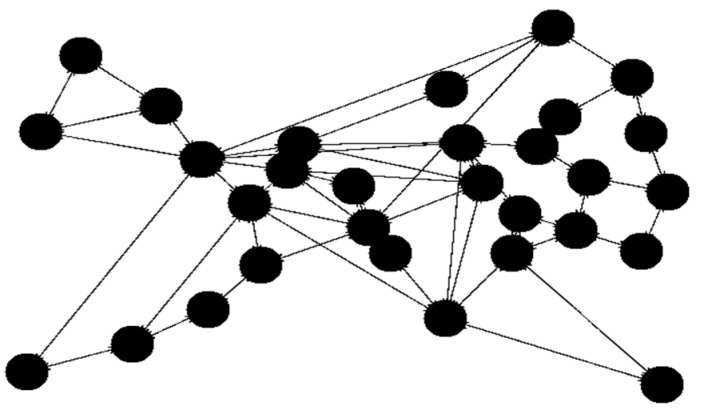
Network topology with 30 nodes (changed topology).

**Figure 13:**
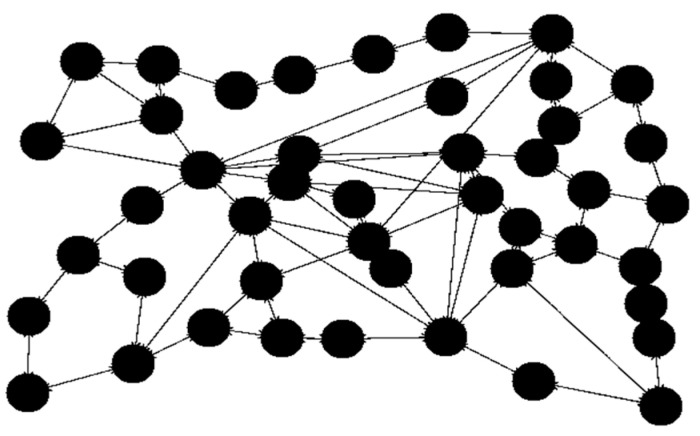
Network topology with 45 nodes had 152 links (changed topology).

**Figure 14:**
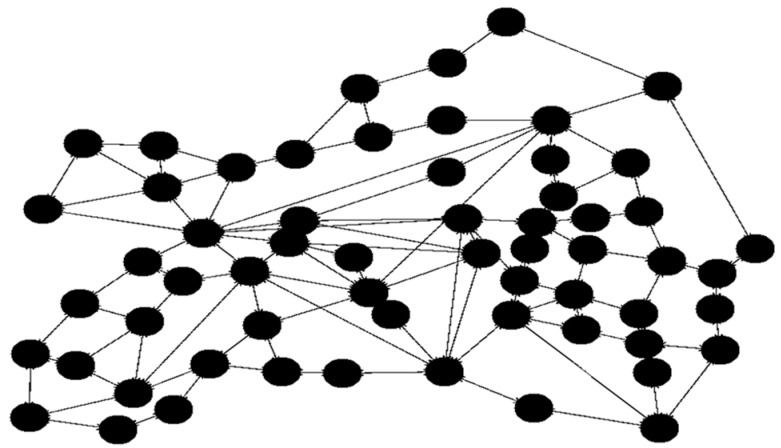
Network topology with 60 nodes (changed topology).

For the second series of tests, the starting point was the built-in network eon_N15_E66 (with 66 links), as depicted in Figure 11. The network was also tested with 30, 45, and 60 nodes five times each, starting from different nodes. Additional nodes and links were randomly added to the basic network. Consequently, the 30-node network (Figure 12) had 108 links, the 45-node network (Figure 13) had 152 links, and the 60-node network (Figure 14) had 214 links. The aim was to significantly increase the number of connections compared to the previous test and evaluate the selected SDN-based energy-saving solutions.

**Figure 15:**
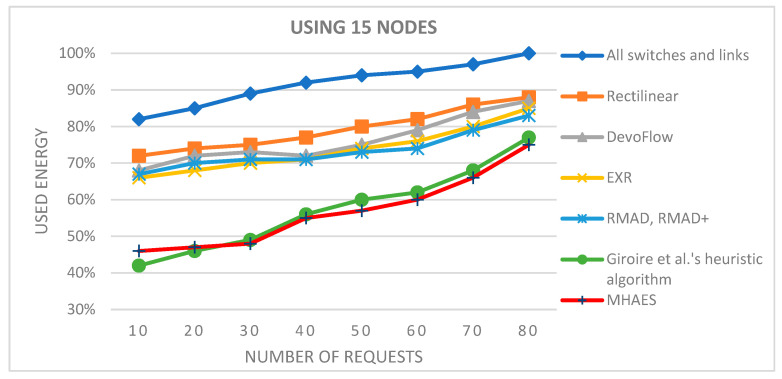
The average energy consumption using 15 nodes depending on the number of requests.

**Figure 16:**
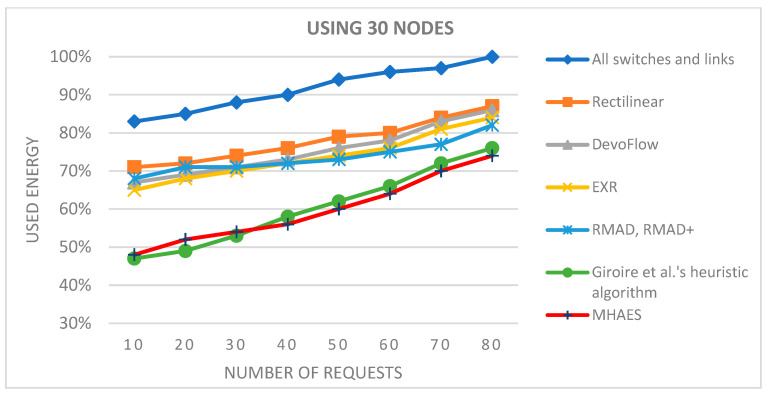
The average energy consumption using 30 nodes depending on the number of requests.

**Figure 17:**
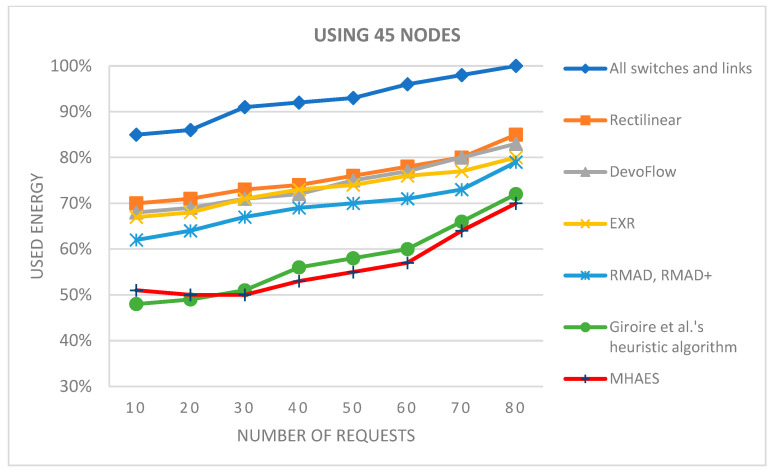
The average energy consumption using 45 nodes depending on the number of requests.

**Figure 18:**
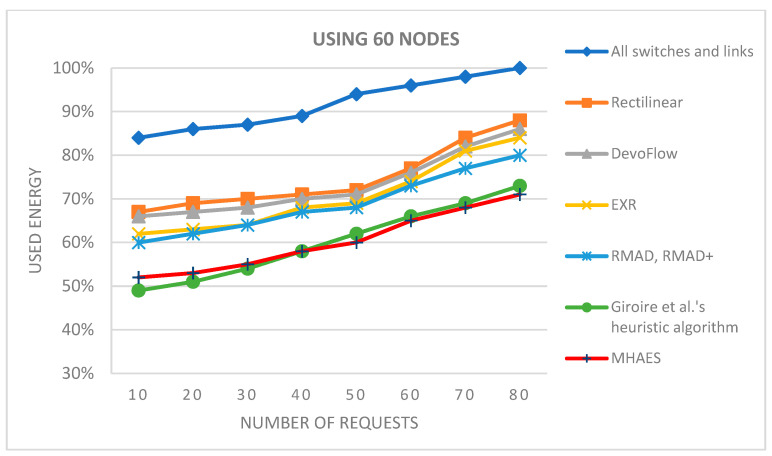
The average energy consumption using 60 nodes depending on the number of requests.

The simulation results are presented in Figure 15, Figure 16, Figure 17 and Figure 18. Similarly to the previous test, the y-axis represents the percentage of energy consumption relative to the expected level, while the x-axis indicates the number of requests. As observed, the network consumed the most energy when all switches and links were active. The average energy savings achieved through the tested methods yielded similar results to the previous topology, although higher link numbers naturally led to increased energy consumption. In the case of 15 nodes (Figure 15), Giroire et al.’s heuristic algorithm and the MHAES yielded the best results. The MHAES achieved the best performance with 25% average savings for 80 requests, surpassing Giroire et al.’s heuristic algorithm by 2%.

For 30 nodes (Figure 16), similarly to the previous topology, the MHAES and Giroire et al.’s heuristic algorithm demonstrated initial energy savings compared to networks with fewer nodes. The MHAES already outperformed the other methods after 35 requests. The energy savings achieved with 80 requests were similar to those obtained with the first topology.

In the case of networks with 45 or 60 nodes (Figure 17 and Figure 18), the MHAES and Giroire et al.’s heuristic algorithm once again outperformed the other tested solutions. For 45 nodes, the MHAES surpassed Giroire et al.’s heuristic algorithm after the 30th request. With 80 requests, these two algorithms still consumed the least energy, but their advantage was not as significant as with a smaller number of nodes. Compared to the RMAD, RMAD+, and Giroire et al.’s heuristic algorithm, the MHAES achieved 7% and 9% less average energy consumption, respectively (compared to all switches and links, the savings were 30%). In the case of 60 nodes, the MHAES outperformed the heuristic algorithm by 2% after 45 requests, maintaining the advantage even after 80 requests. It is important to note that due to the larger number of links, all tested methods exhibited smaller savings compared to the first test series. The deviation from the average value within these topologies in the negative direction was not greater than 1.7% in all cases, while the maximum value in the positive direction was 2.8%. The network performance performed similarly in all cases examined, and no significant differences were observed.

Regarding the MHAES, it is crucial to emphasize that the capacity threshold number and the threshold number of active switches were determined empirically. Further research will focus on predicting these values accurately based on collected samples using fuzzy logic and other artificial intelligence techniques [50,51,52,53]. The development of an effective method to predict the power demand of the network, enabling precise determination of the necessary threshold numbers, is of utmost importance.

## 6. Conclusions

All of the SDN-based energy-saving solutions examined in this study are highly valuable; however, they vary in their energy-saving capabilities. Several studies have been conducted on this topic, producing many relevant findings. Difficulties arose during the investigation due to variations among researchers in terms of the tested methods, network topologies, and software utilized. Therefore, this study conducted two sets of tests, each employing different built-in topologies and four different node numbers per topology. Within these environments, seven different methods were compared. The baseline case involved all switches and links being active, which was then compared to the Rectilinear, DevoFlow, EXR, RMAD, RMAD+, Giroire et al.’s heuristic algorithm solutions, and the MHAES proposed in this article.

Based on the results, it can be concluded that both the proposed MHAES and Giroire et al.’s heuristic algorithm achieved similar performances and provided significantly higher energy savings compared to the other examined solutions. It is worth noting that in all the tested environments, MHAES outperformed Giroire et al.’s heuristic algorithm after 40–45 requests.

To better anticipate future requirements, fuzzy logic can play a crucial role. Leveraging the capabilities of fuzzy logic, it would be advantageous to predict the threshold number for capacity and the number of active switches to be applied in the MHAES, based on network needs and load.

Another aspect that deserves attention in future research is the development and placement of rules on switches. Transferring decision making from the controller to the switches increases the energy consumption of the MHAES for initial requests. Therefore, a suitable solution must be devised to ensure more efficient operation.

Attention must also be paid to the fact that the algorithm has to take into account the diversity of traffic control devices, which can also affect the degree of energy savings.

It would be important to create a fast-intervention SDN application that, based on the queried data, could change the messages sent by the controller with the help of a mobile device of any platform in terms of energy saving [54,55].

## Figures and Tables

**Table 1:** Energy-saving solutions.

Solution Name	Method	Difficulty of Implementation	Network Type
Rectilinear [7]	Compression TCAM solution; rule list	Easy	Fixed network
CAM Razor [25]	Compression TCAM solution—decision diagrams; dynamic programming; redundancy removal	Medium	Fixed network
Compact TCAM [24,25]	Compression TCAM solution—shorter flow entry size	Medium	Fixed network
ECODANE [27]	End Host-Aware solution	Medium	Data center
GAL [9,10]	Energy-aware states; Traffic-Aware	Hard	Fixed network
Exclusive Routing (EXR) [10,30]	Traffic-Aware solution	Medium	Fixed network
SGH [11]	Traffic-Aware solution	Easy	Fixed network
RMAD, RMAD+ [12]	Traffic-Aware; number of active nodes; sleep ratio	Easy	Fixed network
Giroire et al.’s heuristic algorithm [6,13,39]	Rule Placement; TCAM—rule-spaced base; Traffic-Aware	Easy	Fixed network
DevoFlow [40]	Rule Placement; rule cloning; local actions	Easy	Fixed network

**Table 2:** Notation table.

Variable Name	Description
*Z*	SDN switch set
*E*	Link set
*E_ij_*	Link between switches *z_i_* and *z*_j_
*BW_ij_*	Bandwidth of the link between *z_i_* and *z_j_*
*PCS_i_*	Power consumption of switch *z_i_*
*PC_i_*	Power consumption of link *e_ij_*
*f*	Flow of the network
*S_r_, d_s_*	Source switch, destination switch
*f^r^*	Flow rate
*H_in_*	Incoming host
*H_out_*	Outgoing host
*λ_f_*	The packet rate of flow *f*
*L_ij_*	Variable is whether edge *e_ij_* is active or not
*a^f^_i_*	Flow *f* installed on switch *z_i_*
*S_i_*	Status of the switch

**Table 3:** Input variables table.

Variable Name	Description
*Z*	SDN switch set
*E*	Link set
*H*	Hosts
*E_ij_*	Link between switches *z_i_* and *z_j_*
*Bw_ij_*	Bandwidth of the link between *z_i_* and *z_j_*
*R*	Set of offered unicast request
*h_r_, r* ∈ *R*	Offered route rule for *r*. From this information, *s*(*r*) denotes the source host of a demand *r*, and *t*(*r*) is the target host
*E_basic_*	Base power of SDN-enabled switches
*E_configuration_po_*	The power activation of both extreme switches ports of a link. This affects overall *E_configuration_*
*E_configuration_sp_*	Speed rate, this affects overall *E_configuration_*
*E_openflow_control_*	Limited performance associated with control and OpenFlow traffic
*ASW*	Active switches list
*SSW*	Sleeping switches list
*CA*	Maximum capacity of a link
*TCA*	Threshold number for link capacity
*BN*	A big number

**Table 4:** Decision parameters table.

Variable Name	Description
*z_e_*, *e* ∈ *E*	1: if designated link is already installed 0: otherwise (no link means 0 capacity)
*CA_e,_ e* ∈ *E*	The capacity of the designated link
*Y_re_*, *r* ∈ *R*, *e* ∈ *E*	1: if demand *r* passed designated link *e*, 0: otherwise
*y_re_*, *r* ∈ *R*, *e* ∈ *E*	1: if demand *r* passed designated link e, 0: otherwise
*Y_z_*, *z* ∈ *Z*	1: if designated switch is already installed 0: otherwise
*r_z_*, *z* ∈ *Z*	The number of installed OpenFlow flows in designated switch *z*

## Data Availability

The data generated from the simulation is accessible on https://github.com/jzscsaba/MHAES (accessed on 20 October 2023).

## References

[1] Farhady H., Lee H., Nakao A. (2015). Software-defined networking: A survey. Comput. Netw..

[2] Mendiola A., Astorga J., Jacob E., Higuero M. (2016). A survey on the contributions of software-defined networking to traffic engineering. IEEE Commun. Surv. Tutor..

[3] Kannan K., Banerjee S. (2013). Compact TCAM: Flow Entry Compaction in TCAM for Power Aware SDN. Distributed Computing and Networking.

[4] Shirayanagi H., Yamada H., Kono K. Honeyguide: A VM migration-aware network topology for saving energy consumption in data center networks. Proceedings of the 2012 IEEE Symposium on Computers and Communications (ISCC).

[5] Bera S., Misra S., Saha N. (2020). Traffic-Aware Dynamic Controller Assignment in SDN. IEEE Trans. Commun..

[6] Wang L., Li Q., Sinnott R., Jiang Y., Wu J. (2018). An intelligent rule management scheme for Software Defined Networking. Comput. Netw..

[7] Applegate D.L., Calinescu G., Johnson D.S., Karloff H. (2007). Cornpressing rectilinear pictures and rnini rnizing access control lists. ACM-SIAM Syrnposiurn on Discrete Algorithrns (SODA).

[8] Meiners C.R., Liu A.X., Torng E. TCAM Razor: A Systematic Approach Towards Minimizing Packet Classifiers in TCAMs. Proceedings of the 2007 IEEE International Conference on Network Protocols.

[9] Bolla R., Bruschi R., Davoli F., Di Gregorio L., Donadio P., Fialho L., Collier M., Lombardo A., Recupero D.R., Szemethy T. (2013). The Green Abstraction Layer: A Standard Power-Management Interface for Next-Generation Network Devices. IEEE Internet Comput..

[10] Li D., Shang Y., Chen C. Software defined green data center network with exclusive routing. Proceedings of the IEEE INFOCOM 2014—IEEE Conference on Computer Communications.

[11] Markiewicz A., Tran P.N., Timm-Giel A. Energy consumption optimization for software defined networks considering dynamic traffic. Proceedings of the 2014 IEEE 3rd International Conference on Cloud Networking (CloudNet).

[12] Tuysuz M.F., Ankarali Z.K., Gözüpek D. (2017). A survey on energy efficiency in software defined networks. Comput. Netw..

[13] Giroire F., Moulierac J., Phan T.K. Optimizing rule placement in software-defined networks for energy-aware routing. Proceedings of the2014 IEEE Global Communications Conference.

[14] Mogul J.C., Tourrilhes J., Yalagandula P., Sharma P., Curtis A.R., Banerjee S. DevoFlow: Cost-effective flow management for high performance enterprise networks. Proceedings of the 9th ACM SIGCOMM Workshop on Hot Topics in Networks.

[15] Singh S., Jha R.K. (2017). A Survey on Software Defined Networking: Architecture for Next Generation Network. J. Netw. Syst. Manag..

[16] Xia W., Wen Y., Foh C.H., Niyato D., Xie H. (2015). A Survey on Software-Defined Networking. IEEE Commun. Surv. Tutor..

[17] Kreutz D., Ramos F.M.V., Esteves Verissimo P., Rothenberg C.E., Azodolmolky S., Uhlig S. (2015). Software-Defined Networking: A Comprehensive Survey. Proc. IEEE.

[18] Abdelaziz A., Fong A.T., Gani A., Garba U., Khan S., Akhunzada A., Talebian H., Choo K.-K.R. (2017). Distributed controller clustering in software defined networks. PLoS ONE.

[19] Chien W.-C., Lai C.-F., Cho H.-H., Chao H.-C. (2018). A SDN-SFC-based service-oriented load balancing for the IoT applications. J. Netw. Comput. Appl..

[20] Lara A., Kolasani A., Ramamurthy B. (2014). Network Innovation using OpenFlow: A Survey. IEEE Commun. Surv. Tutor..

[21] Li W., Meng W., Kwok L.F. (2016). A survey on OpenFlow-based Software Defined Networks: Security challenges and countermeasures. J. Netw. Comput. Appl..

[22] Kovács M., Agg P.A., Johanyák Z.C. (2020). SDMN Architecture in 5G. Műszaki Tudományos Közlemények.

[23] Pagiamtzis K., Sheikholeslami A. (2006). Content-Addressable Memory (CAM) Circuits and Architectures: A Tutorial and Survey. IEEE J. Solid-State Circuits.

[24] Zhou K.-J., Mu C., Wen B., Zhang X.-M., Wu G.-J., Li C., Jiang H., Xue X.-Y., Tang S., Chen C.-X. (2022). The trend of emerging non-volatile TCAM for parallel search and AI applications. Chip.

[25] Norige E., Liu A.X., Torng E. (2018). A Ternary Unification Framework for Optimizing TCAM-Based Packet Classification Systems. IEEE ACM Trans. Netw..

[26] Agg P., Göcs L., Johanyák Z.C., Borza Z. (2015). Csomagszűrés CISCO routereken ACL-ek segítségével. GRADUS.

[27] Huong T., Schlosser D., Nam P., Jarschel M., Thanh N., Pries R. ECODANE—Reducing energy consumption in data center networks based on traffic engineering. Proceedings of the 11th Würzburg Workshop on IP: Joint ITG and Euro-NF Workshop Visions of Future Generation Networks (EuroView2011).

[28] Heller B., Seetharaman S., Mahadevan P., Yiakoumis Y., Sharma P., Banerjee S., McKeown N. Elastic tree: Saving energy in data center networks. Proceedings of the 7th USENIX Symposium on Networked System Design and Implementation (NSDI).

[29] Keti F., Askar S. Emulation of Software Defined Networks Using Mininet in Different Simulation Environments. Proceedings of the 2015 6th International Conference on Intelligent Systems, Modelling and Simulation.

[30] Adhikari M., Amgoth T. (2018). Heuristic-based load-balancing algorithm for IaaS cloud. Future Gener Comput. Syst..

[31] Govindarajan K., Kumar V.S. An intelligent load balancer for software defined networking (SDN) based cloud infrastructure. Proceedings of the 2017 Second International Conference on Electrical, Computer and Communication Technologies (ICECCT).

[32] Etengu R., Tan S.C., Kwang L.C., Abbou F.M., Chuah T.C. (2020). AI-Assisted Framework for Green-Routing and Load Balancing in Hybrid Software-Defined Networking: Proposal, Challenges and Future Perspective. IEEE Access.

[33] Saha N., Bera S., Misra S. (2021). Sway: Traffic-Aware QoS Routing in Software-Defined IoT. IEEE Trans. Emerg. Top. Comput..

[34] Zhiruo Cao Zheng Wang Zegura E. Rainbow fair queueing: Fair bandwidth sharing without per-flow state. Proceedings of theIEEE INFOCOM 2000, Conference on Computer Communications, Nineteenth Annual Joint Conference of the IEEE Computer and Communications Societies (Cat. No.00CH37064).

[35] Huong T.T., Khoa N.D.D., Dung N.X., Thanh N.H. A global multipath load-balanced routing algorithm based on Reinforcement Learning in SDN. Proceedings of the2019 International Conference on Information and Communication Technology Convergence (ICTC).

[36] Cui J., Lu Q., Zhong H., Tian M., Liu L. (2018). A Load-Balancing Mechanism for Distributed SDN Control Plane Using Response Time. IEEE Trans. Netw. Serv. Manag..

[37] Meng Heang H., Gilani S.M., Hong T., Zhao G., Abdalla H.B. Load Balancing in Wireless Networks using SDN-enabled Infrastructure: Traffic Analysis. Proceedings of the 10th EAI International Conference on Mobile Multimedia Communications.

[38] Diansyah T.M., Handoko D., Faisal I., Yunianti A., Chiuloto K., Liza R. (2019). Design Analysis of OSPF (Open Shortest Path First) Routing by Calculating Packet Loss Of Network WAN (Wide Area Network). J. Phys. Conf. Ser..

[39] Lin W., Zhang L. (2016). The Load Balancing Research of SDN based on Ant Colony Algorithm with Job Classification. Proceedings of the 2016 2nd Workshop on Advanced Research and Technology in Industry Applications.

[40] Curtis A.R., Mogul J.C., Tourrilhes J., Yalagandula P., Sharma P., Banerjee S. DevoFlow: Scaling flow management for high-performance networks. Proceedings of the ACM SIGCOMM 2011 Conference.

[41] Guo Z., Dou S., Wang Y., Liu S., Feng W., Xu Y. (2021). HybridFlow: Achieving Load Balancing in Software-Defined WANs with Scalable Routing. IEEE Trans. Commun..

[42] Ren C., Bai S., Wang Y., Li Y. (2020). Achieving Near-Optimal Traffic Engineering Using a Distributed Algorithm in Hybrid SDN. IEEE Access.

[43] Wei Y., Zhang X., Xie L., Leng S. (2016). Energy-aware traffic engineering in hybrid SDN/IP backbone networks. J. Commun. Netw..

[44] Rout S., Sahoo K.S., Patra S.S., Sahoo B., Puthal D. (2021). Energy Efficiency in Software Defined Networking: A Survey. SN Comput. Sci..

[45] Pap-Szigeti R., Pásztor A. (2021). An examination of skills affecting the effectiveness of programming. Ann. Fac. Engineeting Hunedoara Int. J. Eng..

[46] Csizmás E., Kovács E. (2021). The effect of the dependence structure on risk measures. Gradus.

[47] Pavon-Marino P., Izquierdo-Zaragoza J.-L. (2015). Net2plan: An open source network planning tool for bridging the gap between academia and industry. IEEE Netw..

[48] Jiménez M.D.P., Bueno-Delgado M.V., Pavón-Mariño P. NET2PLAN-UTN: An Educational Tool for Modeling and Planning Urban Transportation Networks. Proceedings of the EDULEARN16 Proceedings.

[49] Kovács T. (2021). Implementing the Intelligent Driver Model in a physical vehicle simulator. Gradus.

[50] Borlea I.-D., Precup R.-E., Borlea A.-B., Iercan D. (2021). A Unified Form of Fuzzy C-Means and K-Means algorithms and its Partitional Implementation. Knowl.-Based Syst..

[51] Blazic S., Dovzan D., Skrjanc I. Cloud-based identification of an evolving system with supervisory mechanisms. Proceedings of the 2014 IEEE International Symposium on Intelligent Control (ISIC).

[52] Vascak J., Kovacik P., Hirota K., Sincak P. Performance-based adaptive fuzzy control of aircrafts. Proceedings of the10th IEEE International Conference on Fuzzy Systems. (Cat. No.01CH37297).

[53] Fogarasi G., Tüü-Szabó B., Földesi P., Kóczy L.T., Cornejo M.E., Kóczy L.T., Medina-Moreno J., Moreno-García J. (2022). Comparison of Discrete Memetic Evolutionary Metaheuristics for TSP. Computational Intelligence and Mathematics for Tackling Complex Problems 2.

[54] Agg P.A., Bolla K.M., Kovács M., Pásztor A. (2021). Natív és cross-platform mobil fejlesztés bemutatása, Demonstration of Genuine Native and Crossplatform Mobile Development. GRADUS.

[55] Kovács M., Johanyák Z.C. (2021). Comparative Analysis of Native and Cross-Platform iOS Application Development. Műszaki Tudományos Közlemények.

